# Supplementary figures and images for: USP4 promotes the proliferation and glucose metabolism of gastric cancer cells by upregulating PKM2
Source: PLoS One. 2023 Aug 25;18(8):e0290688. doi: 10.1371/journal.pone.0290688 (PMC10456134; doi:10.1371/journal.pone.0290688)

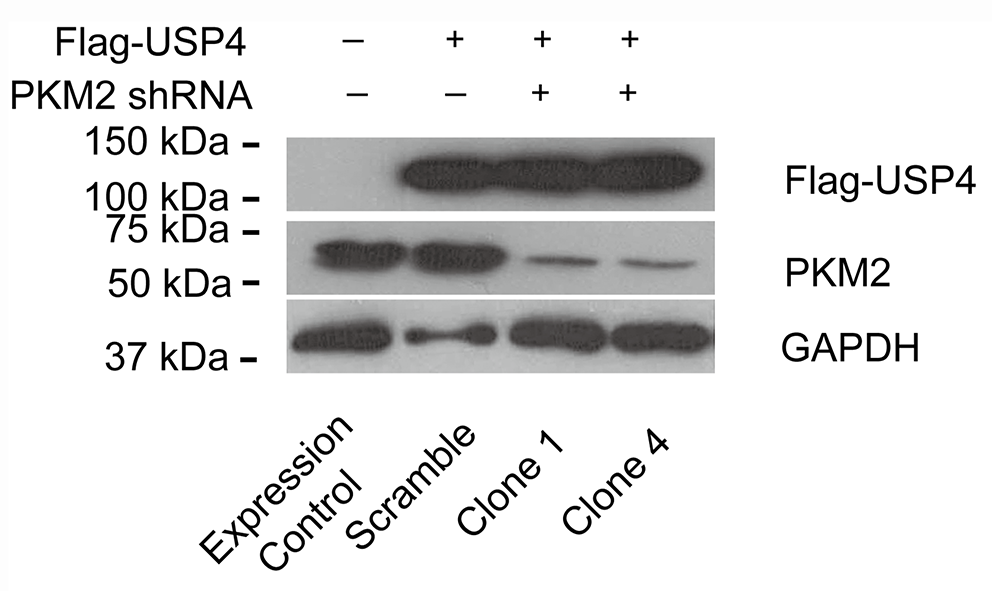

Supplement: S1 Fig — Immunoblotting of USP4, PKM2, and GAPDH in AGS cells that are stably transfected with the USP4 expression plasmid and PKM2 knockdown plasmid, clones 1 and 4, respectively. (TIF) [file pone.0290688.s001.tif]
