# Supplementary material for: USP4 promotes the proliferation and glucose metabolism of gastric cancer cells by upregulating PKM2
Source: PLoS One. 2023 Aug 25;18(8):e0290688. doi: 10.1371/journal.pone.0290688 (PMC10456134; doi:10.1371/journal.pone.0290688)
Supplement: S1 Raw images — (PDF) [file pone.0290688.s002.pdf]

Figs 1A and 1B

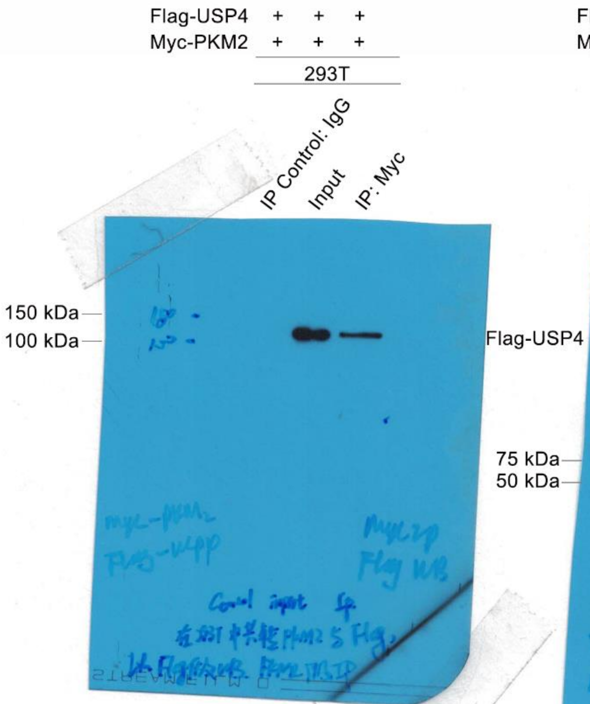

Fig 1A top panel was generated from this original image.

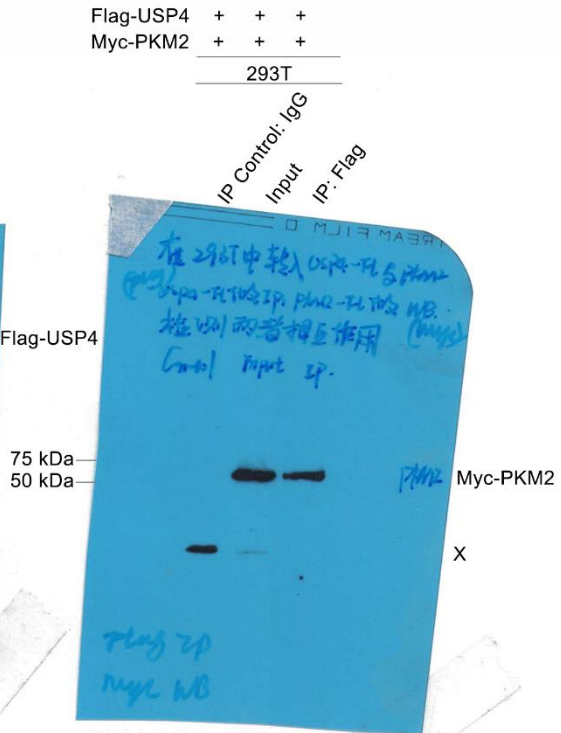

Fig 1A bottom panel was generated from this original image.

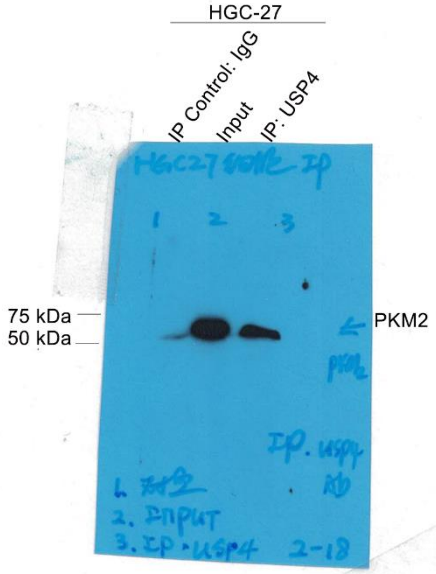

Fig 1B bottom panel was generated from this original image.

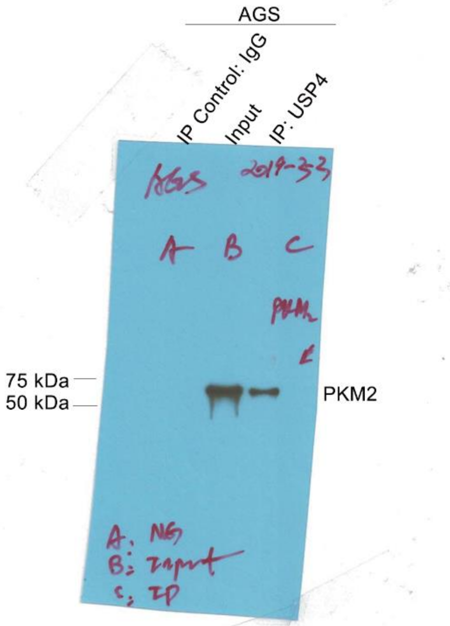

Fig 1B top panel was generated from this original image.

Fig 1C

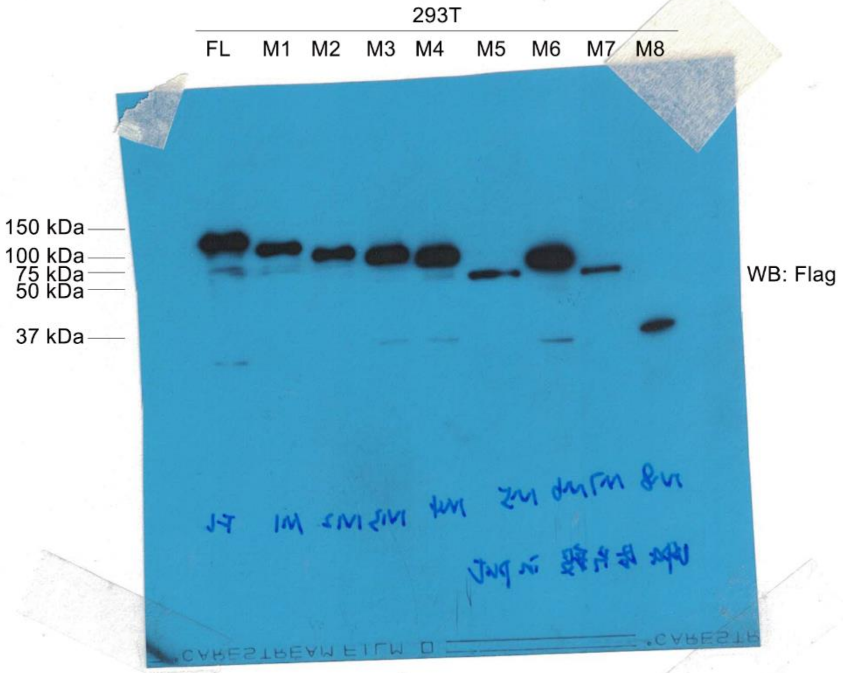

Fig 1C middle panel was generated from this original image.

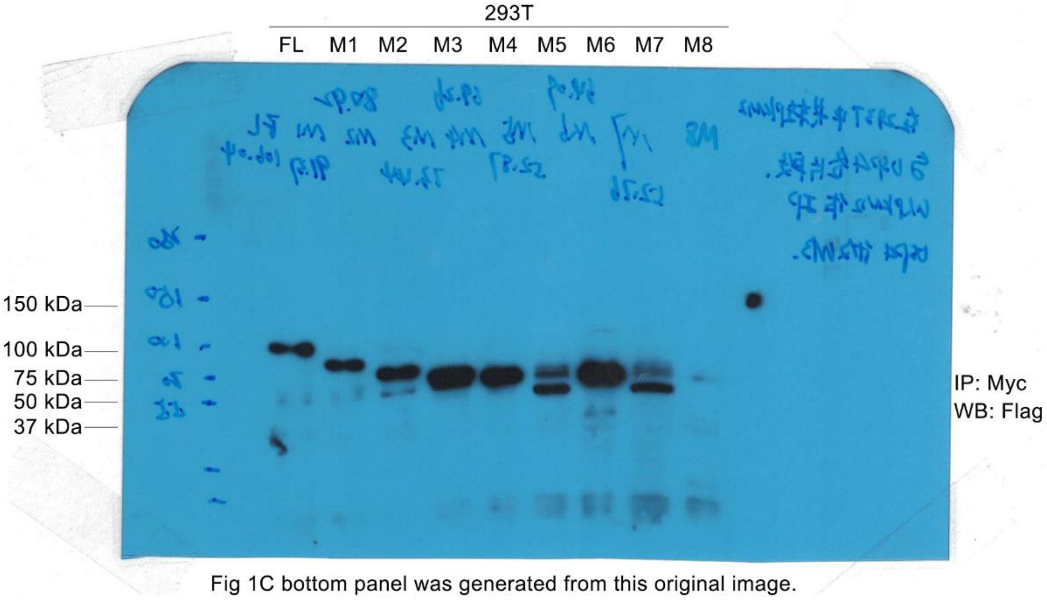

Fig 1C bottom panel was generated from this original image.

Fig 1D

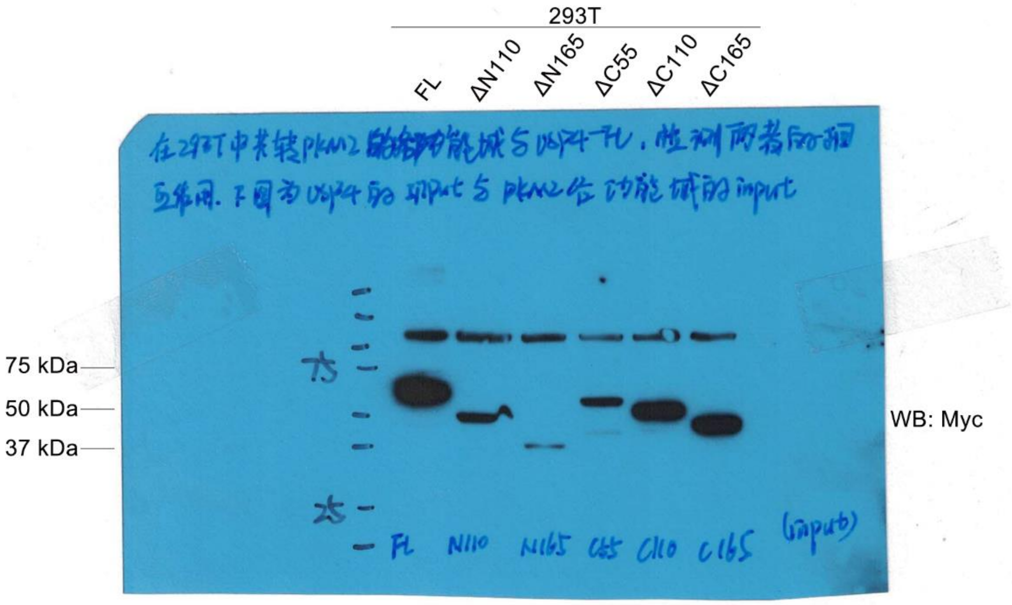

Fig 1D middle panel was generated from this original image.

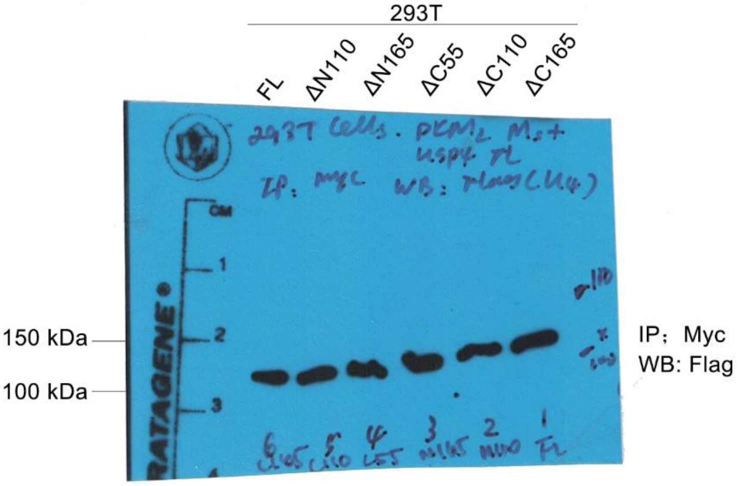

Fig 1D bottom panel was generated from this original image.

Fig 2A

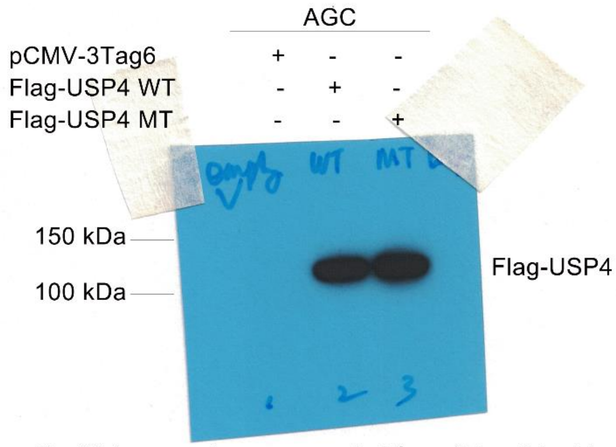

Fig 2A top panel was generated from this original image.

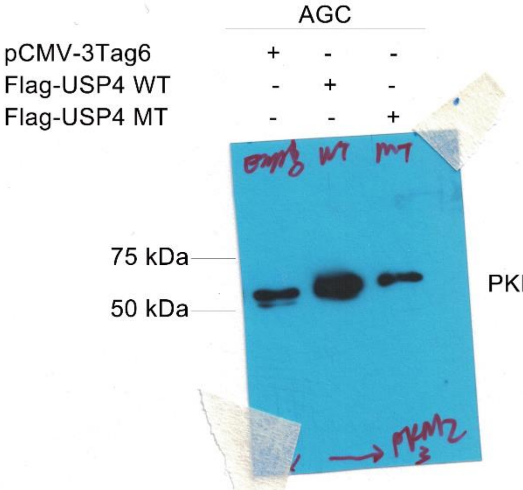

Fig 2A middle panel was generated from this original image.

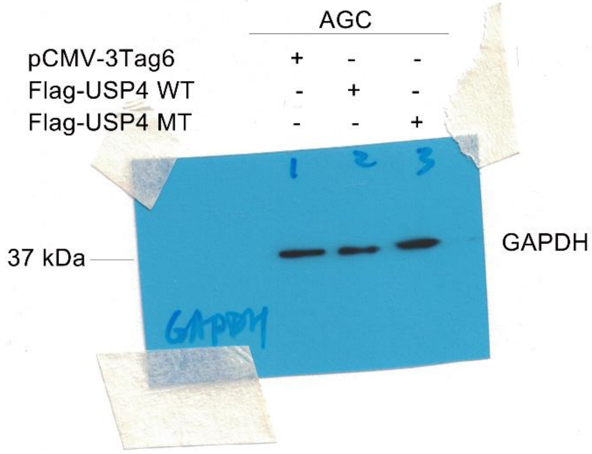

Fig 2A bottom panel was generated from this original image.

Fig 2B

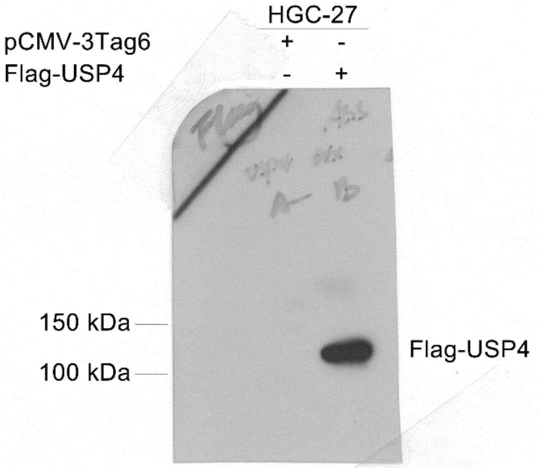

Fig 2B top panel was generated from this original image.

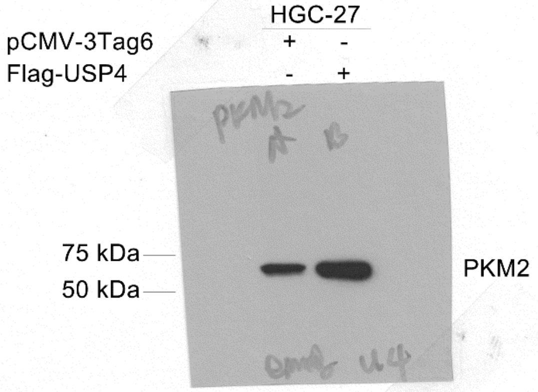

Fig 2B middle panel was generated from this original image.

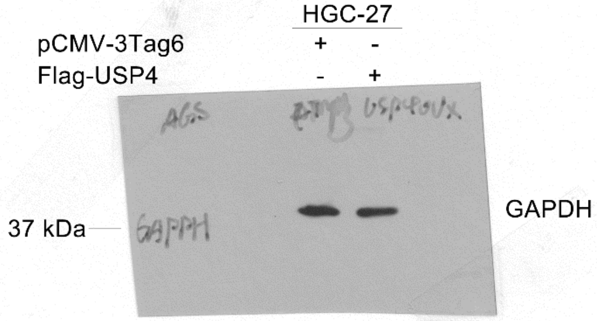

Fig 2B bottom panel was generated from this original image.

Fig 2C

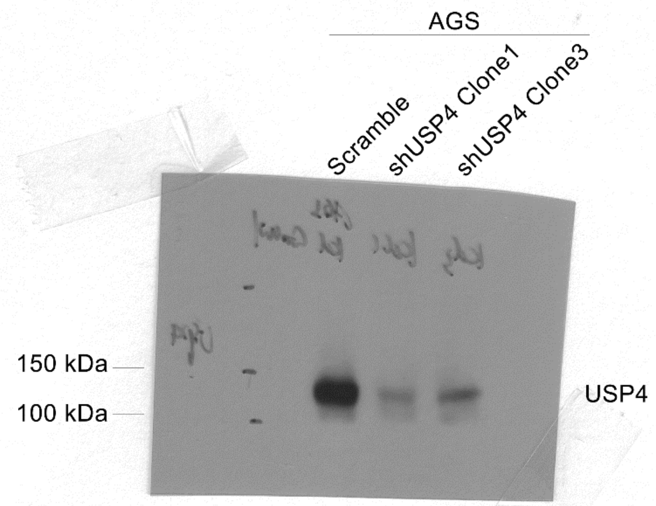

Fig 2C top panel was generated from this original image.

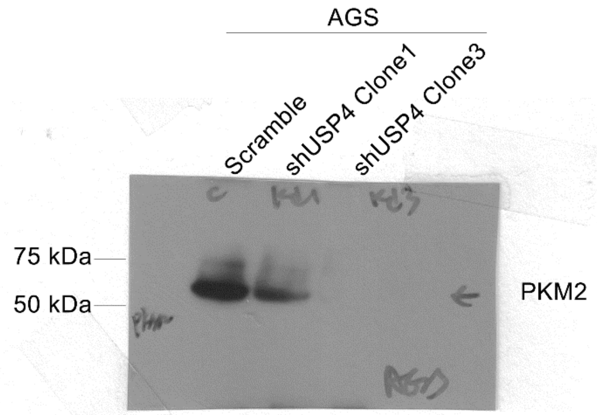

Fig 2C middle panel was generated from this original image.

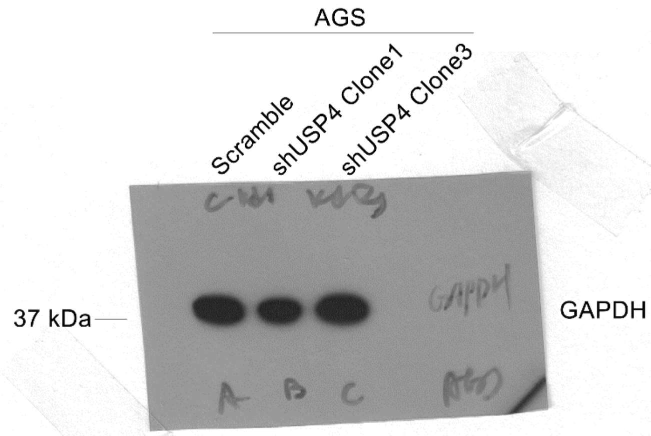

Fig 2C bottom panel was generated from this original image.

Fig 2D

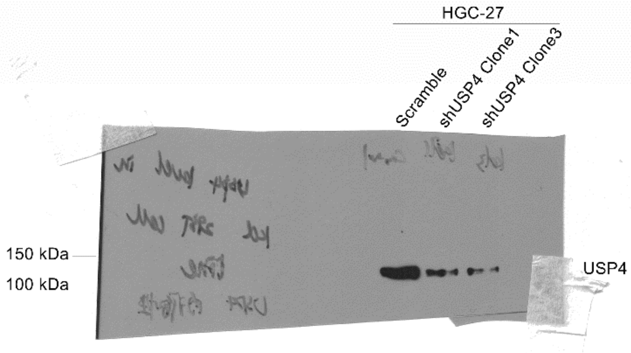

Fig 2D top panel was generated from this original image.

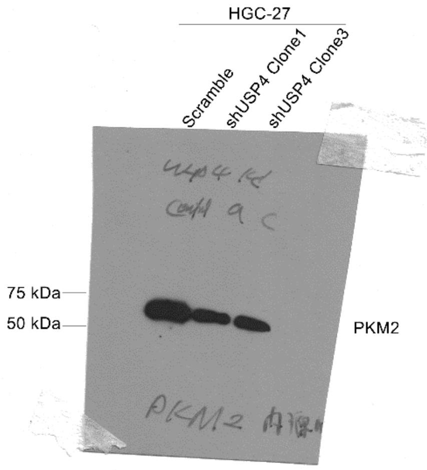

Fig 2D middle panel was generated from this original image.

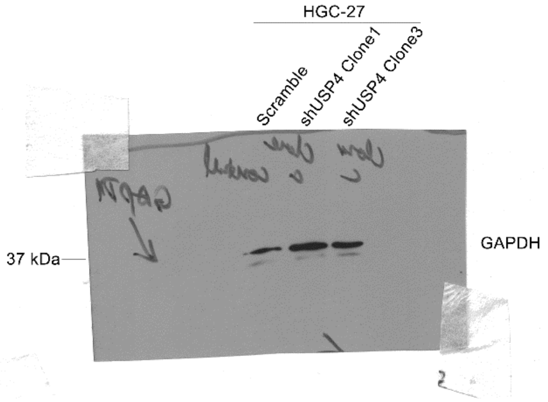

Fig 2D bottom panel was generated from this original image.

Fig 2E

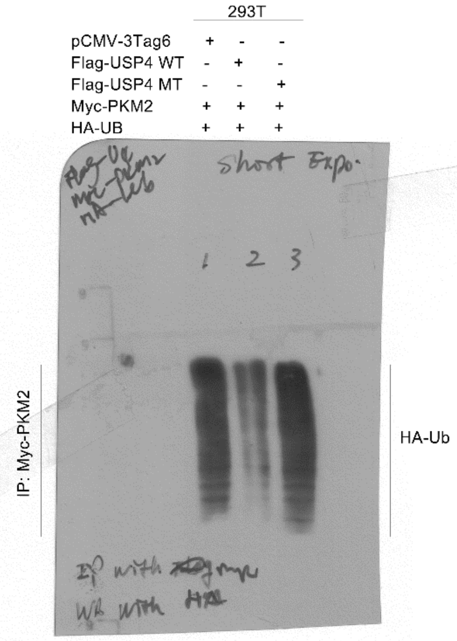

Fig 2E top panel was generated from this original image.

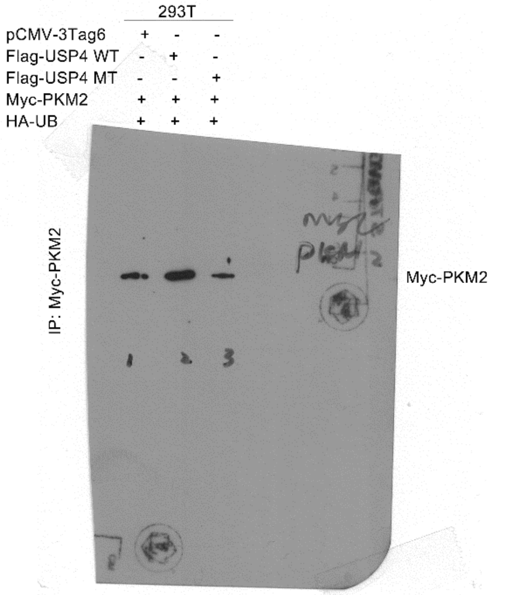

The second panel in Fig 2E was generated from this original image.

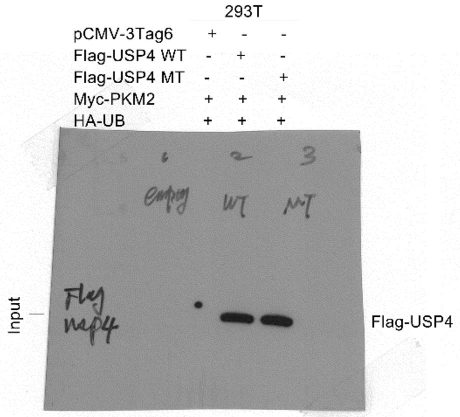

The third panel in Fig 2E was generated from this original image.

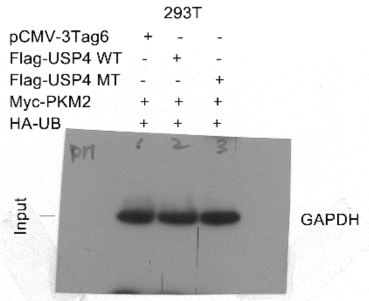

Fig 2E bottom panel was generated from this original image.

S1 Fig

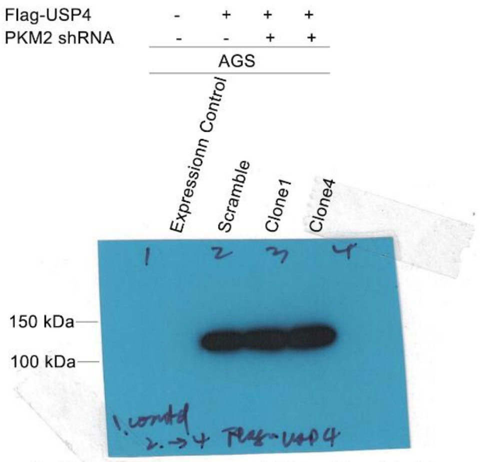

Fig 2D top panel was generated from this original image.

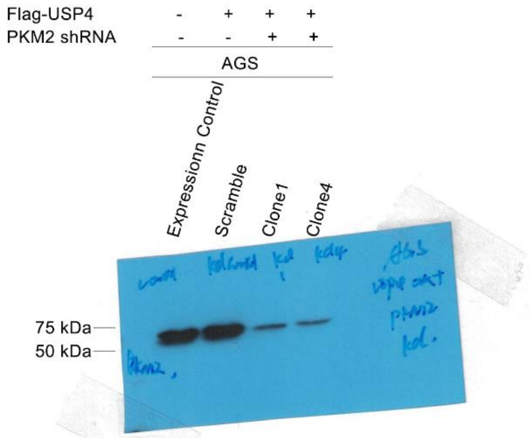

Fig 2D middle panel was generated from this original image.

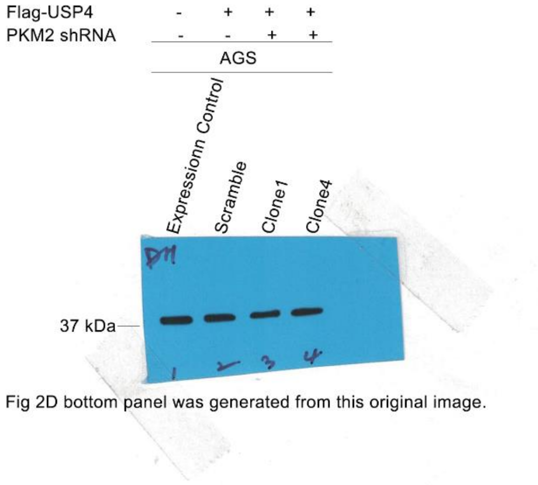

Fig 2D bottom panel was generated from this original image.
