# Supplementary material for: USP4 promotes the proliferation and glucose metabolism of gastric cancer cells by upregulating PKM2
Source: PLoS One. 2023 Aug 25;18(8):e0290688. doi: 10.1371/journal.pone.0290688 (PMC10456134; doi:10.1371/journal.pone.0290688)
Supplement: S1 Table — (PDF) [file pone.0290688.s003.pdf]

Table S1 Primers for human USP4 ORF and deletion mutants

Fragments	Primer Sequences（5'-3'）
USP4-FL	1F(BamHI): ATGGGGATCCATGGCGGAAGGTGGAGGCTGC<br>963R(XhoI): CTAACTCGAGGTTGGTGTCCATGCTGCAAG
USP4-M1(133-963)	133F(BamHI): ATGGGGATCCGTCGAGGTGTATTTGCTGGA<br>963R(XhoI): CTAACTCGAGGTTGGTGTCCATGCTGCAAG
USP4-M2(228-963)	228F(BamHI): ATGGGGATCCACCTTGCAGTCAAAATCAAG<br>963R(XhoI): CTAACTCGAGGTTGGTGTCCATGCTGCAAG
USP4-M3(296-963)	296F(BamHI): ATGGGGATCCCATATACAACCTGGGCTCTGT<br>963R(XhoI): CTAACTCGAGGTTGGTGTCCATGCTGCAAG
USP4-M4(296-925)	296F(BamHI): ATGGGGATCCCATATACAACCTGGGCTCTGT<br>925R(XhoI): CTAACTCGAGATCATCTCGACGTTGGTAAA
USP4-M5(296-776)	296F(BamHI): ATGGGGATCCCATATACAACCTGGGCTCTGT<br>776R(XhoI): CTAACTCGAGCACTGTGGTCTTCTTCTTCT
USP4-M6(296-484+571-963)	296F(BamHI): ATGGGGATCCCATATACAACCTGGGCTCTGT<br>484R(XhoI): CTAACTCGAGCTTCAAGGGCAGTGGCAGCGT<br>571F(XhoI): CTAACTCGAGTCCGTGGATGGCTCGGAATGT<br>963R(BamHI): ATGGGGATCCGTTGGTGTCCATGCTGCAAG
USP4-M7(484-963)	484F(BamHI): ATGGGGATCCAAGAAAGATCGAGTTATGGA<br>963R(XhoI): CTAACTCGAGGTTGGTGTCCATGCTGCAAG
USP4-M8(296-571)	296F(BamHI): ATGGGGATCCCATATACAACCTGGGCTCTGT<br>571R(XhoI): CTAACTCGAGAGTGCTGCAGACCTCGTACAC
USP4-FL(MT)	F: GGGAAACACCGCCTTCATGAACTC<br>R: AGGTTTCCAAGTCCACAG
