# Supplementary material for: USP4 promotes the proliferation and glucose metabolism of gastric cancer cells by upregulating PKM2
Source: PLoS One. 2023 Aug 25;18(8):e0290688. doi: 10.1371/journal.pone.0290688 (PMC10456134; doi:10.1371/journal.pone.0290688)
Supplement: S2 Table — (PDF) [file pone.0290688.s004.pdf]

Table S2 Primers for human PKM2 ORF and deletion mutants

Fragments	Primer Sequences（5’-3’）
PKM2-FL	F(BamHI): ATGGGGATCCATGTCGAAGCCCCATAGTGA
	R(XhoI): CTAACTCGAGTCACGGCACAGGAACAACAC
PKM2-▲N110	F(BamHI): ATGGGGATCCGCTCTAGACACTAAAGGACC
	R(XhoI): CTAACTCGAGTCACGGCACAGGAACAACAC
PKM2-▲N165	F(BamHI): ATGGGGATCCAAGGTGGTGGAAGTGGGCAG
	R(XhoI): CTAACTCGAGTCACGGCACAGGAACAACAC
PKM2-▲C55	F(BamHI): ATGGGGATCCATGTCGAAGCCCCATAGTGA
	R(XhoI): CTAACTCGAGGTCCTTGCACAGCACAGGGA
PKM2-▲C110	F(BamHI): ATGGGGATCCATGTCGAAGCCCCATAGTGA
	R(XhoI): CTAACTCGAGATAAGAAGCCTCCACGCTGCC
PKM2-▲C165	F(BamHI): ATGGGGATCCATGTCGAAGCCCCATAGTGA
	R(XhoI): CTAACTCGAGGGCTGTTTCTCCAGACAGCAT
