# Supplementary material for: USP4 promotes the proliferation and glucose metabolism of gastric cancer cells by upregulating PKM2
Source: PLoS One. 2023 Aug 25;18(8):e0290688. doi: 10.1371/journal.pone.0290688 (PMC10456134; doi:10.1371/journal.pone.0290688)
Supplement: Attachment — Submitted filename: comments to PLOS Yuanyuan et al.docx [file pone.0290688.s005.docx]

In this article, Yuanyuan et al. investigate the unique relationship between USP4 and PKM2 as well as how it controls the way cancer cells use glucose. According to their findings, USP4 controls PKM2 levels through the process of de-ubiquitylation, and its inhibition lowers PKM2 stability. They look at the effects on glucose uptake and metabolism in cancer cells of both over- and under-expressing USP4 and PKM2. They conclude that USP4 modulates PKM2's stability by deubiquitinating, which helps to increase the Warburg effect and support cancer surveillance.

While this paper addresses an essential subject—namely, how USP4 regulates PKM2 levels and their connection to cancer cell metabolism—there are significant concerns that still need to be resolved. These are listed below.

By doing immunoprecipitation with mutants of PKM2 at ubiquitination sites, the findings in Figure 1 may also be significantly reinforced. The available information generally supports the interaction between PKM2 and USP4, although it is not specific to PKM2's ubiquitin state. Additionally, authors can show interaction and localization experiments involving additional cells in Figure 1E.

The authors should use varied expression levels of USP4 and its catalytic mutant to test the stability of PKM2 in a dose-dependent manner.

The authors need to discuss how cells survive when PKM2 levels are significantly reduced (Figure 2C).

When USP4 is overexpressed, the authors should check the levels of ubiquitinated PKM2.

Western blot analysis is necessary to display the PKM2 and USP4 levels in Figures 3 and 4.
